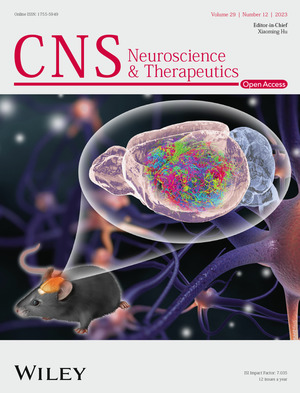# Front cover

**DOI:** 10.1111/cns.14538

**Published:** 2023-11-15

## Abstract

The cover image is based on the Original Article *Whole‐brain monosynaptic inputs to lateral periaqueductal gray glutamatergic neurons in mice* by Wei‐Xiang Ma et al., https://doi.org/10.1111/cns.14338.